# Quantitative Investigation of the Process Parameters of Electrohydrodynamic Direct-Writing and Their Effects on Fiber Surface Roughness and Cell Adhesion

**DOI:** 10.3390/polym12112475

**Published:** 2020-10-25

**Authors:** Chen Jiang, Kan Wang, Xuzhou Jiang, Chuck Zhang, Ben Wang

**Affiliations:** 1 School of Materials Science and Engineering, Georgia Institute of Technology, Atlanta, GA 30332, USA; cjiang74@gatech.edu (C.J.); ben.wang@gatech.edu (B.W.); 2 Georgia Tech Manufacturing Institute, Georgia Institute of Technology, Atlanta, GA 30332, USA; xjiang@gatech.edu (X.J.); chuck.zhang@gatech.edu (C.Z.); 3 H Milton Stewart School of Industrial and System Engineering, Georgia Institute of Technology, Atlanta, GA 30332, USA

**Keywords:** electrohydrodynamic (EHD) direct-writing, electrospinning, surface roughness, cell-substrate interaction, tissue engineering

## Abstract

Electrohydrodynamic (EHD) direct-writing has been widely used to fabricate micro/nanofibers that can serve as a building block in tissue engineering scaffolds. However, the application of EHD direct-writing in tissue engineering is limited by the lack of fundamental knowledge in the correlations among the process parameters, the fiber surface roughness, and the cell adhesion performance. Without a standardized experimental setting and the quantitative database, inconsistent results have been reported. Here, we quantitatively investigate the process–structure–property relationships as the first step towards a better understanding of the EHD direct-writing technology for tissue engineering. Polycaprolactone (PCL) solution is used as a model ink material, and human mesenchymal stem cells (hMSCs) are used to study cell adhesion on PCL fibers. We investigate the different jetting modes defined by the applied voltage, the feed rate, and the nozzle–collector distance. The quantitative effects of process parameters on the fiber surface roughness and the cell adhesion performance are experimentally determined. The quantitative process–structure–property relationships revealed in this study provide guidelines for controlling the surface roughness and the cell adhesion performance of EHD direct-written fibers. This study will facilitate the application of EHD direct-writing in tissue engineering.

## 1. Introduction

Tissue damages caused by diseases or injuries require treatments to facilitate tissue repair, replacement, or regeneration [1]. Organ transplantation remains a major clinical method to repair damaged tissues. However, the shortage of organ donors necessitates tissue engineering development to develop biological substitutes that restore, maintain, and improve the original functionality of damaged tissues [2,3]. One of the biological substitutes’ key elements is a scaffold that provides a suitable environment for cell adhesion, proliferation, and differentiation [4]. Cell–substrate interactions play a crucial role in deciding the scaffolds’ functionality to regulate cellular activities, ranging from attachment and morphology to proliferation and differentiation through contact guidance.

Cell–substrate interactions, relying on a specific binding between the cell membrane’s surface molecules and the substrate, are affected by the physical properties of substrates such as surface roughness, topography, and stiffness [5]. Some of the most commonly used techniques for eliciting the desired cellular responses on biomaterials are photolithography [6] and electron beam lithography [7]. However, these techniques need photosensitive or electrosensitive materials, expensive equipment, and a high level of expertise, hindering the scale up and scale out of these techniques. Low scalability will lead to high costs of tissue engineering products and, subsequently, low patient accessibility of regenerative medicine. Electrospinning is a versatile technique for generating ultrathin fibers with the potential to be specifically engineered to elicit desired cellular responses.

Electrospinning is an electrostatic spinning process that can produce fibers from nearly one hundred different polymers [8,9]. Electrospun fibers have been intensively used in tissue engineering because their fibrous structures mimic the fibrous extracellular matrix [10]. Moreover, the morphology of electrospun fibers can be easily modified to affect cellular activities. The morphology of electrospun fibers can be subdivided into the morphologies of individual fibers and electrospun mats [11].

The morphology of individual fibers can be controlled by changing solvent types [12], collector temperature [13], humidity [12,14,15], or thermal annealing time [16]. For example, Chen et al. have successfully regulated the surface nanoroughness of fibers via humidity control of the electrospinning environment [15]. Results showed that different surface roughnesses supported the expression of different genes. Ribeiro et al. fabricated electrospun fibers with different surface roughness by changing the thermal annealing time and found a higher roughness promoted lower osteoblast but higher fibroblast proliferation [16]. Despite these advantages, the conventional electrospinning process is restricted to applications without the requirement of orderly patterns due to its whipping phenomenon. Most human tissues (e.g., blood vessel, nerve, muscle, etc.) have regular and anisotropic structures. Thus, the morphology of electrospun mats also needs to be modified to facilitate the application of electrospinning in tissue engineering.

The morphology of electrospun mats can be engineered into organized structures using templated collectors [17,18] or electrohydrodynamic (EHD) direct-writing [11,19,20]. Fernandez, P. J. et al. have fabricated electrospun scaffolds with random, radial, and perpendicularly aligned fibers [17]. Results showed that cells adopted different morphologies at different scaffolds, and aligned fibers promoted cell migration. Lee et al. fabricated patterned fibrous mats using EHD direct-writing and realized cell patterning [11].

Desirable cellular responses can be elicited by engineering the morphologies of individual fibers or electrospun mats. In this sense, the morphologies of both individual fibers and electrospun mats can be engineered when fabricating electrospun scaffolds. Zhou et al. fabricated well-aligned electrospun fibers using templated collectors and achieved different surface topography by varying ambient humidity. Results demonstrated a synergistic effect of individual fibers’ morphologies and electrospun mats on cell attachment, proliferation, and alignment [21]. However, this method is limited to aligned fibrous structures, unable to control electrospun scaffolds’ geometric features.

An attractive method to control geometric features of electrospun scaffolds is EHD direct-writing. EHD direct-writing can precisely control the patterning of electrospun fibers by reducing the nozzle-collector distance to eliminate the whipping segment of the electrospun jet. EHD direct-writing has been used to fabricate two-dimensional (2D) [11,22] and three-dimensional (3D) scaffolds [23,24] with the ability to pattern cells. It has also been used to fabricate hybrid scaffolds with improved mechanical properties combined with 3D printing [25]. To achieve better cellular responses, researchers have successfully engineered the morphology of individual fibers in EHD direct-writing, such as the width of fibers [22,26], the straightness of fibers [27,28,29], and the beads on fibers [30], by varying process parameters such as electrical voltage, writing speed, nozzle diameter, and feed rate. However, the effect of process parameters on the surface roughness of EHD direct-written fibers is underinvestigated. Although some studies revealed that the surface roughness of EHD direct-written fibers affected cell attachment and proliferation [24,31,32], quantitative studies about correlations among process parameters, fiber surface roughness, and cell adhesion performance had not been reported for EHD direct-writing so far.

We hypothesize that the surface roughness of the EHD direct-written fiber, and subsequently, the cell adhesion performance can be precisely tuned by controlling the process parameters of EHD direct-writing. We used polycaprolactone (PCL) as the model material for EHD direct-writing. PCL has been widely used in tissue engineering because PCL exhibits reasonable elastic properties and low inflammatory response [33]. PCL and its composites have been widely used in EHD direct-writing [22,23,24]. In this study, we mainly focused on the effect of substrates’ physical properties and did not consider the effect of the chemical properties of substrates on cell adhesion, so we used PCL as the model material in EHD direct-writing.

To test our hypothesis, we conducted three successive tasks. Firstly, we determined the ranges of the process parameters of the EHD direct-writing based on the experimental observation. Secondly, we characterized the surface roughness of EHD direct-written fibers fabricated at different settings of the relevant process parameters. Lastly, we seeded human mesenchymal stem cells (hMSC) on the fibers with different surface roughness and characterized their adhesion performance. The result showed that the fiber surface roughness was affected by the process parameters and cells reacted differently to fibers with different surface roughness. Based on the results, we conclude that our hypothesis is true and report a quantitative guideline to the EHD direct-writing process for tissue engineering. Our findings will facilitate any tissue engineering research using EHD direct-writing as a tool for tissue engineering and enable better manipulation of the scaffold’s physical properties.

## 2. Materials and Methods

### 2.1. Materials

Polycaprolactone (PCL, $M_{n}=80,000$, product number: 440744), Dichloromethane (DCM, product number: 270997), N, N-dimethylformamide (DMF, product number: 227056), and Glutaraldehyde solution (GA, product number: G6257) were purchased from Sigma-Aldrich^®^ (MilliporeSigma, St. Louis, MO, USA). CellTracker™ CM-Dil Dye (category number: C7000) was purchased from Invitrogen (Thermo Fisher Scientific, Waltham, MA, USA). Umbilical cord matrix hMSC (category number: C-12971) and hMSC growth medium (category number: C-28009) were purchased from PromoCell (PromoCell GmbH, Sickingenstr. 63/65, Heidelberg, Germany). Trypsin-EDTA (0.25%, category number: 25200072) was purchased from Gibco™ (Thermo Fisher Scientific, Waltham, MA, USA).

### 2.2. Fiber Fabrication Process

**Figure 1:**
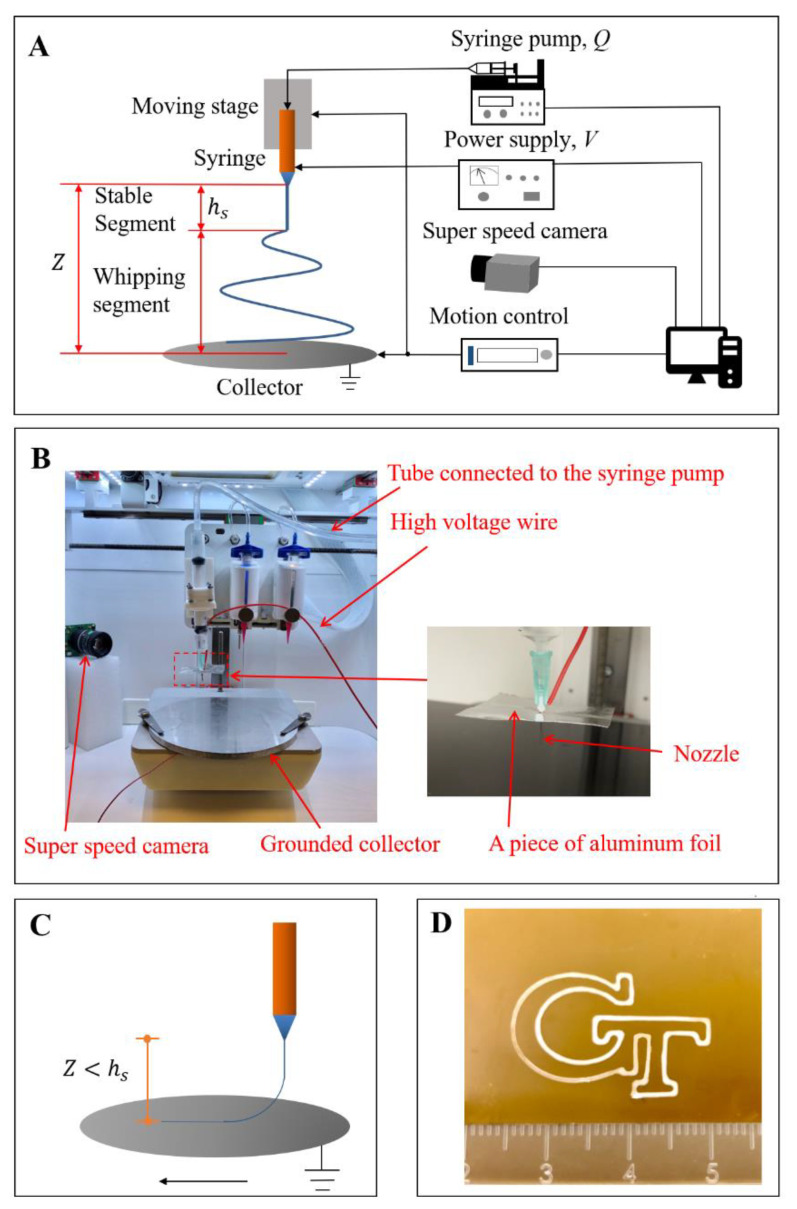
(**A**) Schematic of the electrospinning process. (**B**) The experimental platform (with the amplified image at the nozzle tip). (**C**) Schematic of the EHD direct-writing process. (**D**) ‘GT’ logo fabricated by EHD direct-writing.

Figure 1A,B show the schematic and the experimental setup of electrospinning. We prepared the electrospun solution by dissolving PCL in the DCM:DMF (*v*/*v* ratio = 2:1) cosolvent at a weight concentration of 10%, and then loaded the electrospun solution into a 5 mL syringe, which was connected to a micropump (LEGATO 100, KD Scientific Co., Holliston, MA, USA). The nozzle diameter was 0.8 mm, and the nozzle length was 13 mm. We applied high voltage (PS/FJ30R04.0, Glassman High Voltage Co., High Bridge, NJ, USA) at the nozzle and attached a planar aluminum foil, parallel with the collector, to the nozzle to generate an approximate uniform electric field between the nozzle and the collector (Figure 1B).

To define the process parameters for EHD direct-writing, we selected different settings of the process parameters. Flow rate, *Q*, was set at 30, 35, 40, 45, and 50 μL/min. Nozzle–collector distance, *Z*, was set at 4, 10, 20, 30, 40, and 50 mm. Voltage, *V*, was changed from 3 to 8 kV with an interval of 0.5 kV. We monitored the electrospinning process by a super-speed camera (SMM-C012-U, Mightex Co., Pleasanton, CA, USA). The length of the stable segment of the spun jet, $h_{s}$, was measured by using Image J software (1.8.0, National Institute of Health, Bethesda, MD, USA).

After defining the process parameters for EHD direct-writing (Figure 1C,D), we fabricated EHD direct-written fibers at different process parameters (Table 1) and removed the solvent residue of fibers under vacuum for 24 h at room temperature. The EHD direct-written fibers’ morphologies were examined by a scanning electron microscope (SEM; SU8010, Hitachi, Japan) at 3 kV accelerating voltage after gold-sputtered. The three-dimensional (3D) topography and surface roughness of fibers were characterized by a laser scanning confocal microscope (0L-S40-SU, Olympus^®^ LEXT 3D Material Confocal Microscope, Japan).

### 2.3. Cell Seeding Process

Before seeding cells, we sterilized EHD direct-written fibers by submerging fibers in 75% alcohol for 2 h and then exposing fibers to the ultraviolet light for 30 min. The sterilized fibers were placed in a 24-well plate. The sub-cultured hMSC (P10) were harvested using trypsin-EDTA. A 50 μL hMSC suspension ($5\times 10^{5}$ cells/mL, PromoCell GmbH, Sickingenstr. 63/65, Heidelberg, Germany) was added to each well. After 3 h for cell attachment, we added 3 mL of fresh media into each well. The cell-laden fibers were grown in a 5% CO_2_ incubator at 37 °C, with the medium being replaced every day.

### 2.4. Cell Characterization

After culturing for 3 days, we labeled cells on fibers by using CellTracker^TM^ CM-Dil Dye. We dissolved CM-Dil dye in DMF at 1mg/mL to prepare a stock solution, and then dilute the stock solution into a working solution at a ratio of 1:1000. After washing the cell-laden fibers with phosphate-buffered saline (PBS) for two times, we incubated cell-laden fibers in the working solution for 5 min at 37 °C, and then for an additional 15 min at 4 °C. After labeling, we washed the cell-laden fibers with PBS and observed cells under a fluorescence microscope (BX53, Olympus^®^, Tokyo, Japan). Cell density was calculated using Image J software.

To observe cell morphologies, we submerged the cell-laden fibers in 2.5% GA for 30 min for cell fixation, and then in a series of aqueous alcohol solutions of 30%, 50%, 70%, 90%, and 100% for 10 min, respectively, for dehydration. The samples were gold-sputtered and observed under SEM.

### 2.5. Statistical Analysis

All experiments were performed with five replicates for each sample. The relation between cell density and surface roughness was presented as mean ± standard deviation. Statistical comparisons were performed using Student’s *t*-test. In all analyses, the threshold of *p* values for statistical significance was set to 0.01.

## 3. Results and Discussion

The results were analyzed for the following three purposes: (1) defining the ranges of the process parameters for a steady EHD direct-writing process; (2) investigating the effect of process parameters on the surface roughness of the EHD direct-written fibers; (3) investigating the cell adhesion performance of the EHD direct-written fibers with different roughness. The results and the corresponding analysis are presented in the following subsections.

### 3.1. The Process Parameters Range for a Steady EHD Direct-Writing Process

**Figure 2:**
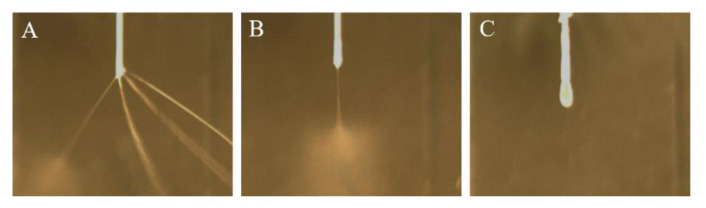
Three working modes of electrospinning process: (**A**) multi-jet mode (*V* = 6 kV, *Q* = 30 μL/min, *Z* = 30 mm), (**B**) single-jet mode (*V* = 4.5 kV, *Q* = 30 μL/min, *Z* = 30 mm), and (**C**) dripping mode (*V* = 3.5 kV, *Q* = 30 μL/min, *Z* = 30 mm).

To define the ranges of the process parameters for a steady EHD direct-writing process, we used different process parameters of electrospinning and observed three different working modes (Figure 2). Figure 2A showed a multi-jet mode since the applied voltage was so high that the cone became unstable [32,34]. Figure 2B showed a stable single-jet mode, which was able to be used for EHD direct-writing. Figure 2C showed a dripping mode, which resulted in the production of beaded fibers or broken fibers [35].

**Figure 3:**
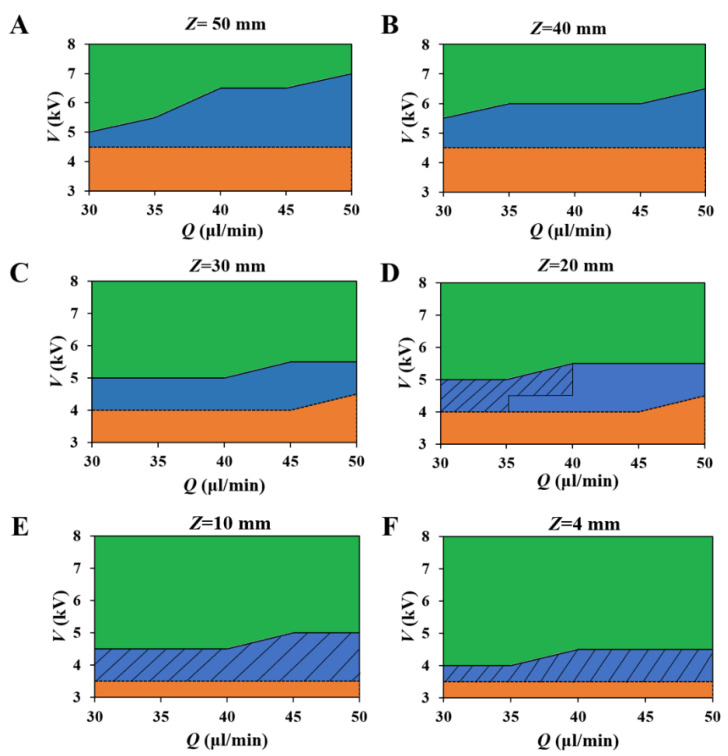
The domains of multi-jet mode (green), single-jet mode (blue), and dripping mode (orange) in the *V*–*Q* parameter plane at different nozzle–collector distance: (**A**) *Z* = 50 mm, (**B**) *Z* = 40 mm, (**C**) *Z* = 30 mm, (**D**) *Z* = 20 mm, (**E**) *Z* = 10 mm, (**F**) *Z* = 4 mm. (The dash areas are EHD direct-writing domains).

**Figure 4:**
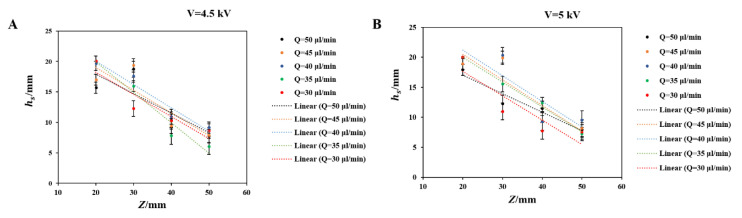
The experimental points and trendlines in the *h_s_*–*Z* plane under different voltage: (**A**) *V* = 4.5 kV, (**B**) *V* = 5 kV.

The mode was affected by the flow rate *Q*, nozzle-collector distance *Z*, and applied voltage *V*. Figure 3 showed the domains of these three electrospinning modes at different *Q*, *Z*, and *V*. The dashed areas were EHD direct-writing domains in which the stable segment length ($h_{s}$) was smaller than *Z*. Since a current breakdown occurred when *Z* was smaller than 4 mm (when *V* was 3.5 kV), the smallest *Z* employed in this study was 4 mm. Figure 4 showed $h_{s}$ at different process parameters and revealed that $h_{s}$ increased with the decrease of *Z*. Thus, a smaller *Z* resulted in a larger fraction of the EHD direct-writing domain in the single-jet mode (Figure 3).

### 3.2. The Effect of Process Parameters on Surface Roughness of EHD Direct-Written Fibers

**Figure 5:**
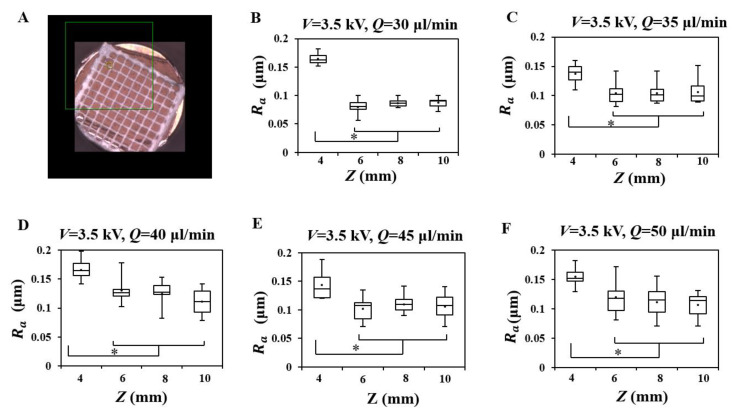
(**A**) Photo of EHD direct-written patterns (10×10 mm square). Roughness ($R_{a}$) at different *Z* when *V* was set at 3.5 kV and *Q* was set at (**B**) 30 μL/min, (**C**) 35 μL/min, (**D**) 40 μL/min, (**E**) 45 μL/min, and (**F**) 50 μL/min (* represents *p* < 0.01).

**Figure 6:**
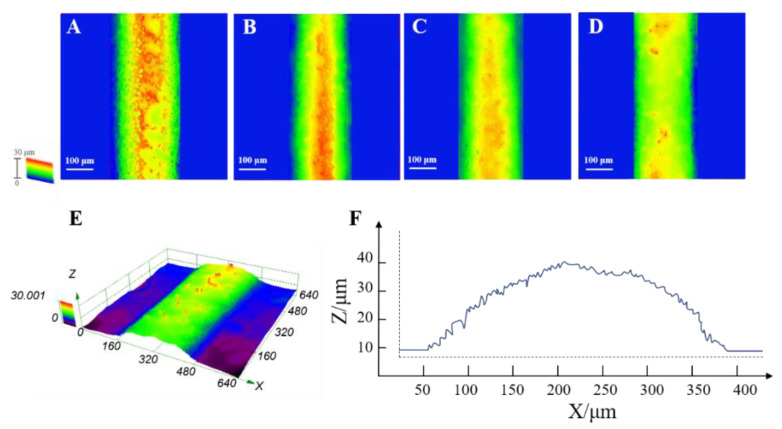
Topographical images captured by the confocal microscopy at 3.5 kV, 30 μL/min, and different *Z*: (**A**) 4 mm, (**B**) 6 mm, (**C**) 8 mm, (**D**) 10 mm. (**E**) The 3D topographical images and (**F**) cross-section of the PCL fiber fabricated when *V* = 3.5 kV, *Q* = 30 μL/min, and *Z* = 4 mm.

**Figure 7:**
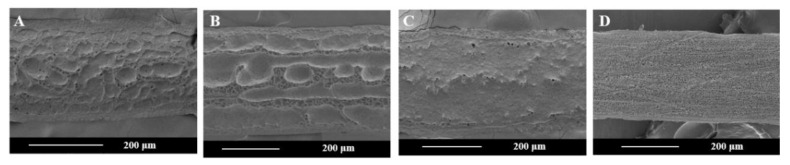
SEM images of fibers fabricated at 3.5 kV, 30 μL/min, and different *Z*: (**A**) 4 mm, (**B**) 6 mm, (**C**) 8 mm, and (**D**) 10 mm.

After defining the ranges of the process parameters for a stable EHD direct-writing process, we fabricated fibers with 10 × 10 mm square patterns by EHD direct-writing (Figure 5A) and characterized the surface roughness ($R_{a}$) of fibers (Figure 6). The results (Figure 5B–F) showed that the $R_{a}$ of fibers fabricated at Z=4 mm was significantly higher than that of other groups. SEM images (Figure 7) showed that the fiber surface changed from more rugged to smoother as *Z* increased from 4 to 10 mm.

Since the small nozzle-collector distance in EHD direct-writing limits solvent evaporation, EHD direct-written fibers remain volatile when deposited. Their morphologies are susceptible to the impact force when deposited on the collector. At the same voltage level, a smaller nozzle-collector distance leads to a larger impact force. Moreover, a smaller nozzle–collector distance is less favorable to solvent evaporation. More solvent left in the deposited fibers makes fiber morphologies more susceptible to the impact force. In this study, fibers fabricated at *Z* = 4 mm had significantly larger surface roughness compared with those fabricated at other *Z*. A threshold of *Z* exists between *Z* = 4 mm and *Z* = 6 mm, which is related to the evaporation rate of the solvent.

### 3.3. The Cell Adhesion Performance of EHD Direct-Written Fibers with Different Roughness

**Figure 8:**
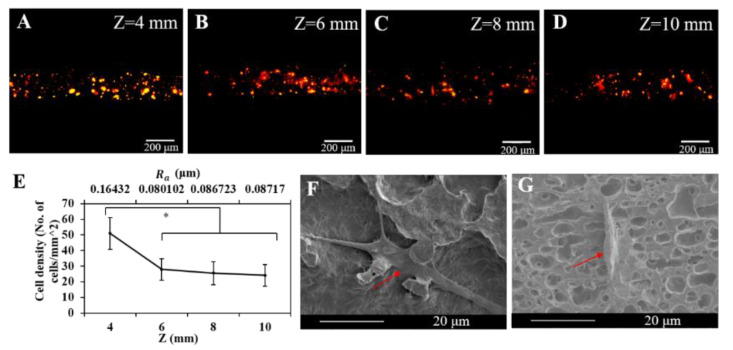
Fluorescence images of cells tracking after culturing the cell-laden samples for 3 days: (**A**) *Z* = 4 mm, (**B**) *Z* = 6 mm, (**C**) *Z* = 8 mm, (**D**) *Z* = 10 mm. (**E**) The correlation between cell density seeded on fibers fabricated at 3.5 kV, 30 μL/min and different *Z* and $R_{a}$ (* represents *p* < 0.01). (**F**) SEM images of cells (red arrow shows) seeded on fibers fabricated at *Z* = 4 mm. (**G**) SEM images of cells (red arrow shows) seeded on fibers fabricated at *Z* = 10 mm.

To investigate the cell adhesion performance of the EHD direct-written fibers with different roughness, we stained cells (Figure 8A–D) and characterized cell density after culturing the cell-laden fibers for 3 days. More cells were attached to fibers fabricated at Z=4 mm with the $R_{a}$ of 0.16 μm than other groups with the $R_{a}$ range of 0.08–0.09 μm (Figure 8E). The result was in agreement with Huang’s research in which he showed that titanium materials with a $R_{a}$ of 0.15 μm achieved the optimal cell adhesion compared to the specimens with either a rougher or smoother surface [36].

**Figure 9:**
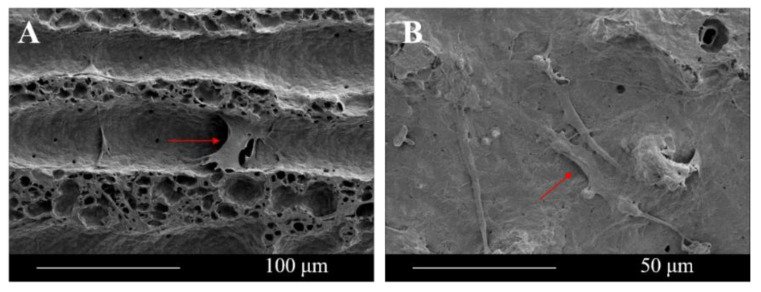
SEM images of cells (red arrow shows) seeded on fibers fabricated at *Z* = 4 mm: (**A**) cross the bump, (**B**) cling on the surface.

Cells exhibited different morphologies on fibers with different roughness. Cells seeded on rugged fibers exhibited the spreading morphology, a sign of good attachment (Figure 8F and Figure 9). In contrast, cells seeded on smoother fibers exhibited spindle morphology, a sign of poor adhesion (Figure 8G) [36,37]. The reason is that fibers with a rougher surface provided more anchor sites for cell adhesion than fibers with a smoother surface did. The adhesion process generated a force on the cytoskeleton, which further affected the following cell activities, including proliferation, apoptosis, and morphology changes.

In this study, EHD direct-written fibers fabricated at Z=4 mm with the $R_{a}$ of 0.16 μm had the largest cell density and the best cell adhesion appearance among all experimental groups. The result indicates that EHD direct-writing can fabricate fibers with different surface roughness for investigating cell-substrate interactions. Various studies have demonstrated that substrates with a specific range of surface roughness selectively enhanced the adhesion, proliferation, or differentiation of a specific type of cells [15,16,38,39]. Therefore, this study brings new insights to design and fabricate EHD direct-written scaffolds with different surface roughness levels for eliciting different cell responses without the necessity of post-processing, which has great potential in connective tissue engineering.

Future studies are needed to investigate long-term cell growth on EHD direct-written scaffolds. Since the hydrophobic nature of PCL is not favorable for long-term cell growth [40,41], hydrophilic materials such as gelatin [42], collagen [43], or chitosan [44] will be considered to add in PCL for EHD direct-writing. Constructing 3D structures using EHD direct-writing will also be explored to investigate the synergistic effect of individual fibers’ morphologies and 3D structures on cell responses.

## 4. Conclusions

In this study, we demonstrated that the surface roughness of the EHD direct-written fibers, and subsequently, the cell adhesion performance could be precisely tuned by controlling the process parameters of EHD direct-writing. We adjusted different process parameters to achieve a stable EHD direct-writing process, and then characterized the surface roughness and cell adhesion performance of the EHD direct-written fibers. The surface roughness of the EHD direct-written fibers was the largest when the *Z* reached the lower limit (4 mm in this study), and biological experiments indicated that the larger roughness was beneficial for cell adhesion. The method and the process–structure–property relationships of EHD direct-writing reported in this study can provide guidelines about how to control the surface roughness and cell adhesion of EHD direct-written fibers, which facilitates the application of EHD direct-writing in tissue engineering.

## Figures and Tables

**Table 1:** The process parameters of EHD direct-writing.

Parameters	Levels				
*Q* (μL/min)	30	35	40	45	50
*Z* (mm)	4	6	8	10	
*V* (kV)	3.5				
Collector moving speed (m/s)	0.1				
